# Supplementary material for: Evaluation of the BD Phoenix Carbapenemase-Producing Organism Panels for the Detection of Carbapenemase Producers in Escherichia coli, Klebsiella pneumoniae and Pseudomonas aeruginosa
Source: Diagnostics (Basel). 2023 Nov 9;13(22):3417. doi: 10.3390/diagnostics13223417 (PMC10670751; doi:10.3390/diagnostics13223417)
Supplement: Supplementary file 1 [file diagnostics-13-03417-s001.zip › diagnostics-2659792-supplementary.pdf]

Supplementary Table S1. Sequence of primers used for PCR reactions.

**Primer name**	**Sequence**
KPC-1	ATGTCACTGTATCGCCGTC
KPC-2	AATCCCTCGAGCGCGAGT
IMP1	CCWGATTTAAAAATYGARAAGCTTG
IMP2	TGGCCAHGCTTCWAHATTTGCRTC
VIM1	GTTTGGTCGCATATCGCAAC
VIM2	AATGCGCAGCACCAGGATAGAA
NDM-F	GGTGCATGCCCGGTGAAATC
NDM-R	ATGCTGGCCTTGGGGAACG
OXA-48A	TTGGTGGCATCGATTATCGG
OXA-48B	GAGCACTTCTTTTGTGATGGC
GES-F	CTATTACTGGCAGGGATCG
GES-R	CCTCTCAATGGTGTGGGT

Supplementary Table S2. CPO report, carbapenemase encoding genes and minimal inhibitory concentration (mg/ml) in *E. coli* clinical isolates

ID	CPO report	*bla*NDM	*bla*KPC	*bla*VIM	*bla*IMP	*bla*OXA-48	AMK	FEP	FOX	CAZ	CRO	CIP	ETP	GEN	IPM	LVX	MEM	MNO	TGC	SXT	SAM	ATM	CZA	CZT	FOS	NIT	NOR	TZP
21-0186	Class A	+	+	-	-	-	≤8	>16	>16	>16	>4	>2	>1	>8	4	>4	>16	2	≤1	>4	>16	>16	4	>8	>128	≤32	>8	>64
21-0044	Class B	+	-	-	-	-	>32	>16	>16	>16	>4	>2	>1	>8	>8	>4	>32	8	≤1	>4	>16	>16	>8	>8	≤16	≤32	>8	>64
21-0054	NR	+	-	-	-	+	≤8	>16	>16	>16	>4	>2	>1	≤2	>8	>4	>32	8	2	>4	>16	>16	>8	>8	≤16	128	>8	>64
21-0058	Class B	+	-	-	-	+	≤8	>16	>16	>16	>4	>2	>1	≤2	>8	>4	>32	>16	2	>4	>16	>16	>8	>8	≤16	≤32	>8	>64
21-0059	NR	+	-	-	-	+	≤8	>16	>16	>16	>4	>2	>1	≤2	>8	>4	>32	8	2	>4	>16	>16	>8	>8	≤16	64	>8	>64
21-0072	Class B	+	-	-	-	+	≤8	>16	>16	>16	>4	>2	>1	>8	>8	>4	>32	4	2	>4	>16	>16	>8	>8	≤16	≤32	>8	>64
21-0080	Class B	+	-	-	-	+	≤8	>16	>16	>16	>4	>2	>1	≤2	>8	>4	>32	4	2	>4	>16	>16	>8	>8	≤16	64	>8	>64
21-0083	NR	+	-	-	-	+	≤8	>16	>16	>16	>4	>2	>1	≤2	>8	>4	>32	8	2	>4	>16	>16	>8	>8	≤16	≤32	>8	>64
21-0085	NR	+	-	-	-	+	8	>16	>16	>16	>4	>2	>1	≤2	>8	>4	>32	8	2	>4	>16	>16	>8	>8	≤16	≤32	>8	>64
21-0090	Class B	+	-	+	-	+	≤8	>16	>16	>16	>4	>2	>1	≤2	>8	>4	>32	4	2	>4	>16	>16	>8	>8	≤16	64	>8	>64
21-0100	NR	-	-	-	-	-	≤8	>16	>16	>16	>4	>2	1	≤2	≤0.25	>4	≤0.5	2	≤1	>4	>16	>16	≤0.25	>8	≤16	≤32	>8	16
21-0103	Class B	+	-	-	-	+	≤8	>16	>16	>16	>4	>2	>1	≤2	>8	>4	>32	4	≤1	>4	>16	>16	>8	>8	≤16	64	>8	>64
21-0108	Class B	+	-	-	-	-	≤8	>16	>16	>16	>4	>2	>1	≤2	>8	>4	>32	4	2	>4	>16	>16	>8	>8	≤16	≤32	>8	>64
21-0118	NR	+	-	+	-	+	≤8	>16	>16	>16	>4	>2	>1	≤2	>8	>4	>32	8	2	>4	>16	>16	>8	>8	≤16	64	>8	>64
21-0119	Class B	+	-	+	-	+	≤8	>16	>16	>16	>4	>2	>1	≤2	>8	>4	>32	4	2	>4	>16	>16	>8	>8	≤16	64	>8	>64
21-0131	NR	+	-	-	-	+	≤8	>16	>16	>16	>4	>2	>1	≤2	>8	>4	>32	16	2	>4	>16	>16	>8	>8	≤16	≤32	>8	>64
21-0133	NR	+	-	-	-	+	≤8	>16	>16	>16	>4	>2	>1	≤2	>8	4	>32	8	2	>4	>16	>16	>8	>8	≤16	64	>8	>64
21-0134	Class B	+	-	-	-	+	≤8	>16	>16	>16	>4	>2	>1	≤2	>8	>4	>32	4	2	>4	>16	>16	>8	>8	≤16	64	>8	>64
21-0177	Class B	+	-	-	-	+	≤8	>16	>16	>16	>4	>2	>1	≤2	>8	>4	>32	4	2	>4	>16	>16	>8	>8	≤16	64	>8	>64
21-0181	CP	+	-	-	-	+	8	>16	>16	>16	>4	>2	>1	>8	>8	>4	>32	≤1	≤1	>4	>16	>16	>8	>8	≤16	≤32	>8	>64
21-0182	Class B	+	-	-	-	+	≤8	>16	>16	>16	>4	>2	>1	≤2	>8	>4	>32	8	2	>4	>16	>16	>8	>8	≤16	≤32	>8	>64
21-0183	NR	+	-	-	-	-	≤8	>16	>16	>16	>4	>2	>1	≤2	>8	>4	32	8	≤1	>4	>16	>16	>8	>8	≤16	≤32	>8	>64
21-0184	Class B	+	-	-	-	+	≤8	>16	>16	>16	>4	>2	>1	≤2	>8	>4	32	8	2	>4	>16	>16	>8	>8	≤16	64	>8	>64
21-0185	Class B	+	-	-	-	-	8	>16	>16	>16	>4	>2	>1	>8	>8	>4	>32	≤1	≤1	>4	>16	>16	>8	>8	≤16	≤32	>8	>64
21-0187	Class B	+	-	-	-	+	≤8	>16	>16	>16	>4	>2	>1	≤2	>8	>4	>32	16	2	>4	>16	>16	>8	>8	≤16	≤32	>8	>64
21-0191	Class B	+	-	-	-	+	8	>16	>16	>16	>4	>2	>1	≤2	>8	>4	32	4	2	>4	>16	>16	>8	>8	≤16	≤32	>8	>64
21-0192	Class B	+	-	-	-	+	≤8	>16	>16	>16	>4	>2	>1	≤2	>8	>4	32	4	2	>4	>16	>16	>8	>8	≤16	≤32	>8	>64
21-0196	Class B	+	-	-	-	-	≤8	>16	>16	>16	>4	>2	>1	≤2	>8	>4	>32	8	4	>4	>16	>16	>8	>8	64	>128	>8	>64
21-0197	NR	-	-	-	-	-	≤8	>16	>16	>16	>4	>2	>1	>8	0.5	>4	≤0.5	8	≤1	≤1	>16	>16	>8	>8	≤16	≤32	>8	>64
21-0198	Class D	-	-	-	-	-	≤8	>16	>16	>16	>4	>2	>1	≤2	>8	>4	>32	2	≤1	>4	>16	>16	≤0.25	>8	≤16	≤32	>8	>64
21-0199	NR	+	-	-	-	-	≤8	>16	>16	>16	>4	>2	>1	≤2	1	>4	2	16	≤1	>4	>16	>16	>8	4	≤16	≤32	>8	16
21-0201	NR	-	-	-	-	+	≤8	>16	>16	>16	>4	>2	>1	≤2	>8	>4	32	8	2	>4	>16	>16	>8	>8	≤16	≤32	>8	>64
21-0203	Class B	+	-	-	+	+	≤8	>16	>16	>16	>4	>2	>1	≤2	>8	>4	>32	4	≤1	>4	>16	>16	>8	>8	≤16	≤32	>8	>64
21-0210	Class B	+	-	-	-	+	≤8	>16	>16	>16	>4	>2	>1	≤2	>8	>4	32	4	2	>4	>16	>16	>8	>8	≤16	128	>8	>64
21-0212	NR	+	-	-	-	-	≤8	>16	>16	>16	>4	>2	>1	≤2	>8	>4	>32	8	2	>4	>16	>16	>8	>8	≤16	64	>8	>64
21-0213	Class B	+	-	-	-	+	≤8	>16	>16	>16	>4	>2	>1	≤2	>8	>4	>32	4	2	>4	>16	>16	>8	>8	≤16	≤32	>8	>64
21-0215	NR	+	-	-	-	+	≤8	>16	>16	>16	>4	>2	>1	≤2	>8	>4	>32	8	2	>4	>16	4	>8	>8	≤16	128	>8	>64
21-0216	NR	+	-	-	-	+	≤8	>16	>16	>16	>4	>2	>1	≤2	>8	>4	32	4	≤1	2	>16	>16	>8	>8	≤16	64	>8	>64
21-0217	Class B	+	-	-	-	+	≤8	>16	>16	>16	>4	>2	>1	≤2	>8	>4	32	4	2	>4	>16	>16	>8	>8	≤16	≤32	>8	>64
21-0219	Class B	-	+	-	-	+	≤8	>16	>16	>16	>4	>2	>1	≤2	>8	>4	32	4	2	>4	>16	>16	>8	>8	≤16	128	>8	>64
21-0236	Class B	-	-	-	-	-	≤8	>16	>16	>16	>4	>2	>1	>8	>8	>4	16	4	2	>4	>16	>16	>8	>8	≤16	≤32	>8	>64
21-0240	Class B	+	-	+	-	+	8	>16	>16	>16	>4	>2	>1	≤2	>8	>4	>32	16	2	>4	>16	>16	>8	>8	≤16	128	>8	>64
21-0284	Class B	+	-	+	-	-	≤8	>16	>16	>16	>4	>2	>1	≤2	>8	>4	>32	8	≤1	>4	>16	>16	>8	>8	≤16	≤32	>8	>64
21-0287	NR	-	-	-	-	-	≤8	>16	>16	>16	>4	>2	>1	≤2	>8	>4	32	8	2	>4	>16	>16	>8	>8	≤16	64	>8	>64
21-0288	Class B	+	-	-	-	-	≤8	>16	>16	>16	>4	>2	>1	≤2	>8	4	8	≤1	≤1	≤1	>16	>16	8	>8	≤16	≤32	4	>64
21-0289	NR	+	-	-	-	+	≤8	>16	>16	>16	>4	>2	>1	≤2	>8	4	32	8	4	>4	>16	>16	>8	>8	≤16	>128	>8	>64
21-0301	NR	+	-	-	-	+	≤8	>16	>16	>16	>4	>2	>1	≤2	>8	>4	32	8	4	>4	>16	>16	>8	>8	≤16	≤32	>8	>64
21-0302	Class B	+	-	-	-	+	≤8	>16	>16	>16	>4	>2	>1	≤2	>8	>4	>32	4	≤1	>4	>16	>16	>8	>8	≤16	≤32	>8	>64
21-0350	NR	+	-	-	-	+	≤8	>16	>16	>16	>4	>2	>1	≤2	>8	>4	>32	8	2	>4	>16	>16	>8	>8	≤16	≤32	>8	>64
21-0622	NR	+	-	-	-	+	≤8	>16	>16	>16	>4	>2	>1	≤2	>8	>4	>16	8	2	>4	>16	>16	>8	>8	≤16	≤32	>8	>64
21-0623	Class B	+	-	-	-	+	≤8	>16	>16	>16	>4	>2	>1	≤2	>8	>4	>16	4	≤1	>4	>16	>16	>8	>8	≤16	64	>8	>64
21-0624	Class B	+	-	-	-	+	≤8	>16	>16	>16	>4	>2	>1	≤2	>8	>4	32	4	2	>4	>16	>16	>8	>8	≤16	64	>8	>64
21-0625	Class B	+	-	-	-	+	≤8	>16	>16	>16	>4	>2	>1	≤2	>8	>4	>16	4	≤1	>4	>16	>16	>8	>8	≤16	64	>8	>64
21-0627	Class B	+	-	-	-	+	≤8	>16	>16	>16	>4	>2	>1	≤2	>8	>4	>16	8	2	>4	>16	>16	>8	>8	≤16	64	>8	>64
21-0628	Class B	+	-	-	-	+	8	>16	>16	>16	>4	>2	>1	≤2	>8	>4	32	8	2	>4	>16	>16	>8	>8	>128	≤32	>8	>64
21-0630	NR	+	-	-	-	+	≤8	>16	>16	>16	>4	>2	>1	≤2	>8	>4	32	8	2	>4	>16	>16	>8	>8	≤16	64	>8	>64
21-0792	Class B	+	-	-	-	+	≤8	>16	>16	>16	>4	>2	>1	≤2	>8	>4	16	4	2	>4	>16	>16	>8	>8	≤16	≤32	>8	>64
21-0793	Class B	+	-	-	-	+	≤8	>16	>16	>16	>4	>2	>1	≤2	>8	>4	32	2	≤1	>4	>16	>16	>8	>8	≤16	≤32	>8	>64
21-0795	Class B	+	-	-	-	+	≤8	>16	>16	>16	>4	>2	>1	≤2	>8	>4	16	4	≤1	>4	>16	>16	>8	>8	≤16	≤32	>8	>64
21-0796	Class B	+	-	+	-	+	≤8	>16	>16	>16	>4	>2	>1	≤2	>8	>4	>32	8	2	>4	>16	>16	>8	>8	≤16	≤32	>8	>64
21-0797	NR	+	-	-	-	-	≤8	>16	>16	>16	>4	>2	>1	≤2	>8	>4	>16	8	≤1	>4	>16	>16	>8	>8	≤16	≤32	>8	>64
21-0826	NR	+	-	-	-	+	≤8	>16	>16	>16	>4	>2	>1	≤2	>8	>4	>32	4	2	>4	>16	>16	>8	>8	≤16	≤32	>8	>64
21-0827	NR	+	-	-	-	+	≤8	>16	>16	>16	>4	>2	>1	≤2	4	>4	8	4	2	>4	>16	>16	>8	>8	≤16	≤32	>8	>64
21-0828	Class B	+	-	-	-	+	≤8	>16	>16	>16	>4	>2	>1	≤2	>8	>4	>32	4	≤1	>4	>16	>16	>8	>8	≤16	≤32	>8	>64
21-0829	Class B	+	-	-	-	+	≤8	>16	>16	>16	>4	>2	>1	≤2	>8	>4	32	8	2	>4	>16	>16	>8	>8	≤16	64	>8	>64
21-0833	NR	+	-	-	-	+	≤8	>16	>16	>16	>4	>2	>1	≤2	>8	>4	>32	4	2	>4	>16	>16	>8	>8	≤16	≤32	>8	>64
21-1017	NR	-	-	-	-	-	32	>16	>16	>16	>4	>2	>1	>8	>8	>4	32	8	≤1	>4	>16	>16	4	>8	≤16	≤32	>8	>64
21-1022	Class D	-	-	-	-	-	≤8	8	>16	2	>4	>2	>1	>8	1	>4	≤0.5	16	4	>4	>16	8	0.5	2	≤16	≤32	>8	64
21-1023	NR	-	-	-	-	-	≤8	>16	>16	>16	>4	>2	>1	≤2	>8	>4	>32	8	2	2	>16	>16	>8	>8	≤16	64	>8	>64
21-1025	NR	-	-	-	-	-	≤8	>16	>16	>16	>4	2	>1	≤2	>8	≤1	16	2	≤1	>4	>16	>16	>8	>8	≤16	≤32	4	64
21-1026	NR	-	-	-	-	-	≤8	>16	>16	>16	>4	2	>1	≤2	>8	≤1	32	≤1	≤1	>4	>16	>16	>8	>8	≤16	≤32	4	>64
21-1031	Class B	+	-	-	-	-	≤8	>16	>16	>16	>4	>2	>1	≤2	>8	>4	>16	4	≤1	>4	>16	>16	>8	>8	≤16	≤32	>8	>64
21-1034	NR	-	-	-	-	-	≤8	8	>16	8	>4	>2	>1	>8	≤0.25	>4	1	4	2	>4	8	>16	2	4	128	≤32	>8	>64
21-1042	NR	-	-	-	-	-	8	>16	>16	>16	>4	>2	>1	≤2	>8	>4	>32	8	2	>4	>16	>16	>8	>8	≤16	≤32	>8	>64
21-1046	NR	-	-	-	-	-	≤8	>16	>16	>16	>4	>2	>1	≤2	>8	>4	>32	8	2	>4	>16	>16	>8	>8	≤16	64	>8	>64
21-1057	Class B	-	-	-	-	-	8	>16	>16	>16	>4	>2	>1	≤2	>8	>4	32	8	2	>4	>16	>16	>8	>8	≤16	≤32	>8	>64
22-1162	NR	-	-	-	-	-	≤8	>16	>16	>16	>4	>2	>1	≤2	>8	>4	32	8	2	>4	>16	>16	>8	>8	≤16	≤32	>8	>64
22-1191	NR	-	-	-	-	-	8	>16	16	>16	>4	>2	1	>8	≤0.25	>4	≤0.5	2	≤1	>4	>16	>16	0.5	≤1	≤16	≤32	>8	16
22-1221	NR	-	-	-	-	-	≤8	>16	>16	>16	>4	>2	>1	≤2	>8	>4	>32	4	4	>4	>16	>16	>8	>8	64	>128	>8	>64

22-1222	Class B	+	-	-	-	-	8	>16	>16	>16	>4	>2	>1	≤2	>8	>4	>32	4	2	>4	>16	>16	>8	>8	≤16	≤32	>8	>64
22-1230	Class B	-	-	-	-	-	≤8	>16	>16	>16	>4	>2	>1	≤2	>8	>4	>32	4	2	>4	>16	>16	>8	>8	≤16	64	>8	>64
22-1282	Class B	+	-	-	-	-	≤8	>16	>16	>16	>4	>2	>1	≤2	>8	>4	32	8	2	>4	>16	>16	>8	>8	≤16	128	>8	>64
22-1297	NR	-	-	-	-	-	>32	>16	>16	>16	>4	>2	>1	>8	>8	>4	8	8	2	>4	>16	>16	>8	>8	≤16	≤32	>8	>64
22-1336	Class B	+	-	-	-	-	>32	>16	>16	>16	>4	>2	>1	>8	>8	>4	8	8	2	>4	>16	>16	>8	>8	≤16	≤32	>8	>64
22-1374	Class B	+	-	-	-	-	≤8	>16	>16	>16	>4	>2	>1	≤2	>8	>4	16	4	≤1	>4	>16	>16	>8	>8	≤16	64	>8	>64
22-1393	NR	-	-	-	-	+	≤8	>16	>16	>16	>4	>2	>1	≤2	2	>4	1	>16	≤1	>4	>16	>16	4	>8	≤16	≤32	>8	>64
22-1396	NR	-	-	-	-	+	≤8	>16	>16	>16	>4	>2	>1	≤2	2	>4	1	>16	≤1	>4	>16	>16	4	>8	≤16	≤32	>8	>64
22-1399	Class D	-	-	-	-	+	≤8	>16	>16	>16	>4	>2	>1	>8	1	>4	1	4	≤1	≤1	>16	>16	4	>8	≤16	≤32	>8	>64
22-1401	Class B	+	-	-	-	-	≤8	>16	>16	>16	>4	>2	>1	≤2	>8	>4	>32	8	2	>4	>16	>16	>8	>8	≤16	64	>8	>64
22-1412	Class B	+	-	-	-	-	≤8	>16	>16	>16	>4	>2	>1	≤2	>8	>4	>32	8	2	>4	>16	>16	>8	>8	≤16	64	>8	>64
22-1413	Class B	+	-	-	-	-	≤8	>16	>16	>16	>4	>2	>1	≤2	>8	>4	>32	8	2	>4	>16	>16	>8	>8	≤16	≤32	>8	>64
22-1419	Class B	+	-	-	-	-	≤8	>16	>16	>16	>4	>2	>1	≤2	>8	>4	>32	8	2	>4	>16	>16	>8	>8	≤16	64	>8	>64
22-1431	NR	+	-	-	-	-	≤8	>16	>16	>16	>4	>2	>1	≤2	>8	>4	>32	16	4	>4	>16	>16	>8	>8	≤16	≤32	>8	>64
22-1438	NR *	-	-	-	-	+	>32	>16	16	>16	>4	>2	>1	>8	1	>4	≤0.5	8	≤1	>4	>16	>16	1	4	≤16	≤32	>8	64
22-1439	NR *	-	-	-	-	+	>32	>16	16	>16	>4	>2	>1	>8	1	>4	1	8	2	>4	>16	>16	0.5	4	≤16	≤32	>8	64
22-1441	NR	+	-	-	-	-	≤8	>16	>16	>16	>4	>2	>1	≤2	>8	>4	32	8	2	>4	>16	>16	>8	>8	≤16	64	>8	>64
22-1691	NR	+	-	-	-	-	≤8	>16	>16	>16	>4	>2	>1	≤2	>8	>4	>32	8	2	>4	>16	>16	>8	>8	≤16	64	>8	>64
22-1698	Class B	+	-	-	-	-	≤8	>16	>16	>16	>4	>2	>1	≤2	>8	>4	>32	8	2	>4	>16	>16	>8	>8	≤16	≤32	>8	>64
22-1699	NR	+	-	-	-	-	≤8	>16	>16	>16	>4	>2	>1	≤2	>8	>4	32	4	≤1	>4	>16	>16	>8	>8	≤16	≤32	>8	>64
22-1710	NR	-	-	-	-	-	≤8	>16	>16	>16	>4	>2	>1	≤2	>8	>4	>32	8	2	>4	>16	>16	>8	>8	≤16	64	>8	>64
22-1737	Class B	+	-	-	-	-	≤8	>16	>16	>16	>4	>2	>1	≤2	>8	>4	>32	16	2	>4	>16	>16	>8	>8	≤16	≤32	>8	>64
22-1776	NR	+	-	-	-	-	≤8	>16	>16	>16	>4	>2	>1	≤2	>8	>4	2	8	2	>4	>16	>16	>8	>8	≤16	64	>8	>64
22-1780	Class B	+	-	-	-	-	≤8	>16	>16	>16	>4	>2	>1	≤2	>8	>4	>32	8	2	>4	>16	>16	>8	>8	≤16	128	>8	>64
22-1782	NR	-	-	-	-	-	8	>16	>16	>16	>4	>2	1	4	≤0.25	>4	≤0.5	16	2	>4	>16	>16	8	>8	≤16	128	>8	>64
22-1804	Class B	+	-	-	-	-	>32	>16	>16	>16	>4	>2	>1	>8	>8	>4	>32	4	≤1	>4	>16	16	>8	>8	≤16	≤32	>8	>64
22-1808	Class B	+	-	-	-	-	8	>16	>16	>16	>4	>2	>1	≤2	>8	>4	>32	2	≤1	>4	>16	>16	>8	>8	64	≤32	>8	>64
22-1809	Class B	+	-	-	-	-	8	>16	>16	>16	>4	>2	>1	≤2	>8	>4	>32	2	≤1	>4	>16	>16	>8	>8	32	≤32	>8	>64
22-1810	Class B	+	-	-	-	-	≤8	>16	>16	>16	>4	>2	>1	≤2	>8	>4	>32	4	2	>4	>16	>16	>8	>8	≤16	≤32	>8	>64
22-1832	Class B	+	-	+	-	-	≤8	>16	>16	>16	>4	>2	>1	≤2	>8	>4	>32	4	2	>4	>16	>16	>8	>8	≤16	≤32	>8	>64
22-1833	Class B	+	-	-	-	-	≤8	>16	>16	>16	>4	>2	>1	≤2	>8	>4	>16	4	2	>4	>16	>16	>8	>8	≤16	≤32	>8	>64
22-1834	NR	+	-	-	-	-	≤8	>16	>16	>16	>4	>2	>1	≤2	>8	>4	>16	4	2	>4	>16	>16	>8	>8	≤16	≤32	>8	>64
22-1855	Class B	+	-	-	-	-	≤8	>16	>16	>16	>4	>2	>1	≤2	>8	>4	>32	8	2	>4	>16	>16	>8	>8	≤16	128	>8	>64
22-1856	NR	+	-	-	-	-	8	>16	>16	>16	>4	>2	>1	≤2	>8	>4	>32	8	2	>4	>16	>16	>8	>8	≤16	>128	>8	>64
22-1860	Class B	+	-	-	-	-	≤8	>16	>16	>16	>4	>2	>1	≤2	>8	>4	>32	8	2	>4	>16	>16	>8	>8	≤16	128	>8	>64
22-1862	NR	+	-	-	-	-	≤8	>16	>16	>16	>4	>2	>1	≤2	>8	>4	>32	8	2	>4	>16	>16	>8	>8	≤16	≤32	>8	>64
22-1863	Class B	+	-	-	-	-	≤8	>16	>16	>16	>4	>2	>1	≤2	>8	>4	32	>16	2	>4	>16	>16	>8	>8	≤16	≤32	>8	>64
22-1864	NR	+	-	-	-	-	≤8	>16	>16	>16	>4	>2	>1	≤2	>8	>4	>32	8	2	>4	>16	>16	>8	>8	≤16	≤32	>8	>64
22-1865	NR	+	-	-	-	-	≤8	>16	>16	>16	>4	>2	>1	≤2	>8	>4	>32	8	2	>4	>16	>16	>8	>8	≤16	≤32	>8	>64
22-1866	Class B	+	-	-	-	-	≤8	>16	>16	>16	>4	>2	>1	≤2	>8	>4	>32	4	2	>4	>16	>16	>8	>8	≤16	≤32	>8	>64
22-1880	Class B	+	-	-	-	-	≤8	>16	>16	>16	>4	>2	>1	≤2	>8	>4	>32	8	2	>4	>16	>16	>8	>8	≤16	≤32	>8	>64
22-1881	Class B	+	-	-	-	-	≤8	>16	>16	>16	>4	>2	>1	≤2	>8	>4	>32	8	2	>4	>16	>16	>8	>8	≤16	≤32	>8	>64
22-1882	NR	+	-	-	-	-	≤8	>16	>16	>16	>4	>2	>1	≤2	>8	>4	>32	8	2	>4	>16	>16	>8	>8	≤16	≤32	>8	>64
22-1937	Class B	+	-	-	-	-	≤8	>16	>16	>16	>4	>2	>1	≤2	>8	>4	32	4	2	>4	>16	>16	>8	>8	≤16	≤32	>8	>64
22-1938	Class B	+	-	-	-	-	≤8	>16	>16	>16	>4	>2	>1	≤2	>8	>4	>32	4	≤1	>4	>16	>16	>8	>8	≤16	≤32	>8	>64
22-1941	Class B	+	-	-	-	-	≤8	>16	>16	>16	>4	>2	>1	≤2	>8	>4	32	4	≤1	≤1	>16	>16	>8	>8	≤16	≤32	>8	>64
22-1949	CP	-	-	-	-	+	≤8	≤1	16	≤2	≤1	1	>1	≤2	4	≤1	4	≤1	≤1	≤1	>16	≤1	≤0.25	>8	≤16	≤32	8	>64
22-1950	NR	+	-	-	-	+	≤8	>16	>16	>16	>4	>2	>1	≤2	>8	>4	32	8	≤1	>4	>16	>16	>8	>8	≤16	64	>8	>64
22-1952	NR	+	-	-	-	-	≤8	>16	>16	>16	>4	>2	>1	≤2	>8	>4	16	8	2	>4	>16	2	>8	>8	≤16	≤32	>8	>64
22-1953	Class B	+	-	-	-	-	≤8	>16	>16	>16	>4	>2	>1	≤2	>8	>4	>32	4	≤1	>4	>16	≤1	>8	>8	≤16	≤32	>8	>64
22-1954	Class B	+	-	-	-	-	≤8	>16	>16	>16	>4	>2	>1	≤2	>8	>4	16	8	2	>4	>16	>16	>8	>8	≤16	64	>8	>64
22-1963	Class B	+	-	-	-	-	≤8	>16	>16	>16	>4	>2	>1	≤2	>8	>4	32	4	≤1	>4	>16	>16	>8	>8	≤16	64	>8	>64
22-1987	Class B	+	-	-	-	-	≤8	>16	>16	>16	>4	>2	>1	≤2	>8	>4	>32	4	2	>4	>16	>16	>8	>8	≤16	≤32	>8	>64
22-1988	Class B	+	-	-	-	-	≤8	>16	>16	>16	>4	>2	>1	≤2	>8	>4	16	4	≤1	>4	>16	>16	8	>8	≤16	64	>8	>64
22-1992	Class B	+	-	-	-	-	≤8	>16	>16	>16	>4	>2	>1	≤2	>8	>4	16	8	2	>4	>16	>16	>8	>8	≤16	≤32	>8	>64
22-2012	NR	-	-	-	-	-	8	>16	>16	>16	>4	>2	1	>8	1	>4	≤0.5	8	2	≤1	>16	>16	>8	>8	≤16	≤32	>8	>64
22-2021	Class B	+	-	-	-	-	≤8	>16	>16	>16	>4	>2	>1	≤2	>8	>4	>32	8	2	>4	>16	>16	>8	>8	≤16	128	>8	>64
22-2024	Class B	+	-	-	-	-	≤8	>16	>16	>16	>4	>2	>1	≤2	>8	>4	>32	8	≤1	>4	>16	>16	>8	>8	≤16	≤32	>8	>64
22-2091	Class B	+	-	-	-	-	≤8	>16	>16	>16	>4	>2	>1	≤2	>8	>4	>16	4	≤1	>4	>16	>16	>8	>8	≤16	64	>8	>64
22-2092	Class B	+	-	-	-	-	8	>16	>16	>16	>4	≤0.12	>1	≤2	>8	≤1	32	2	≤1	>4	>16	>16	8	>8	64	≤32	≤2	>64
22-2113	Class B	-	-	+	-	+	≤8	2	8	≤2	>4	0.5	>1	>8	4	≤1	1	≤1	≤1	>4	>16	8	8	≤1	≤16	≤32	≤2	64
22-2114	Class B	+	-	-	-	-	≤8	>16	>16	>16	>4	>2	>1	≤2	>8	>4	>16	4	2	>4	>16	>16	>8	>8	≤16	64	>8	>64
23-2126	Class B	+	-	-	-	-	≤8	>16	>16	>16	>4	>2	>1	≤2	>8	>4	32	4	≤1	>4	>16	>16	>8	>8	≤16	≤32	>8	>64
23-2127	Class B	+	-	-	-	-	≤8	>16	>16	>16	>4	>2	>1	≤2	>8	>4	>32	4	2	>4	>16	>16	>8	>8	≤16	≤32	>8	>64
23-2244	Class B	+	-	+	-	-	≤8	>16	>16	>16	>4	>2	>1	≤2	>8	>4	>32	>16	2	>4	>16	>16	>8	>8	≤16	128	>8	>64
23-2245	Class B	+	-	-	-	-	8	>16	>16	>16	>4	>2	>1	≤2	>8	>4	32	4	≤1	>4	>16	>16	>8	>8	≤16	≤32	>8	>64
23-2262	Class B	+	-	-	-	-	≤8	>16	>16	>16	>4	>2	>1	≤2	>8	>4	>32	8	2	>4	>16	>16	>8	>8	≤16	>128	>8	>64
23-2313	Class B	+	-	-	-	-	≤8	>16	>16	>16	>4	>2	>1	≤2	>8	>4	>32	8	2	>4	>16	>16	>8	>8	≤16	128	>8	>64
23-2324	Class B	+	-	+	-	-	≤8	>16	>16	>16	>4	>2	>1	≤2	>8	>4	>32	8	2	>4	>16	>16	>8	>8	≤16	64	>8	>64
23-2325	Class B	+	-	-	-	-	≤8	>16	>16	>16	>4	>2	>1	≤2	>8	>4	>32	8	2	>4	>16	>16	>8	>8	≤16	64	>8	>64
23-2373	Class B	+	-	-	-	-	≤8	>16	>16	>16	>4	>2	>1	≤2	>8	>4	>32	8	2	>4	>16	>16	>8	>8	≤16	64	>8	>64

AMK: amikacin, FEP: cefepime, FOX, cefoxitin, CAZ: ceftazidime, CRO: ceftriaxone, CIP: ciprofloxacin, ETP: ertapenem, GEN: gentamicin, IPM: imipenem, LVX: levofloxacin, MEM: meropenem, MNO: minocycline, TGC: tigecycline, SXT: trimethoprim/sulfamethoxazole, SAM: ampicillin/sulbactam, ATM: aztreonam, CZA: ceftazidime-avibactam, CZT: ceftolozane/tazobactam, FOS: fosfomycin, NIT: nitrofurantoin, NOR: norfloxacin, TZP: piperacillin/tazobactam. NR: no recommendation, CP: carbapenemase producer. All had a MIC for ampicillin>16, cefuroxime XM >16, colistin ≤1. * Extended spectrum β–lactamase producer. – negative, + positive

Supplementary Table S3. CPO report, carbapenemase encoding genes and minimal inhibitory concentration (μg/ml) in *K. pneumoniae* clinical isolates.

ID	CPO report	*bla*NDM	*bla*KPC	*bla*VIM	*bla*IMP	*bla*OXA-48	AMK	FEP	FOX	CAZ	CRO	CIP	COL	ETP	GEN	IPM	LVX	MEM	MNO	TGC	SXT	SAM	ATM	CZA	CZT	CXM	FOS	NIT	NOR	TZP
21-0194	Class A	-	+	-	-	-	≤8	>16	16	>16	>4	≤0.12	≤1	>1	≤2	>8	≤1	32	2	2	≤1	>16	>16	4	>8	>16	≤16	64	≤2	>64
22-1271	Class B	-	-	-	-	-	16	>16	>16	>16	>4	>2	≤1	>1	4	>8	>4	32	>16	4	>4	>16	>16	1	>8	>16	≤16	>128	>8	>64
21-0001	Class B	+	-	-	-	-	>32	>16	>16	>16	>4	2	≤1	>1	>8	>8	≤1	16	4	2	>4	>16	>16	>8	>8	>16	>128	64	4	>64
21-0003	NR	+	-	-	-	-	32	>16	>16	>16	>4	>2	≤1	>1	>8	>8	>4	>32	16	4	>4	>16	>16	>8	>8	>16	≤16	128	>8	>64
21-0004	Class B	+	-	-	-	+	>32	>16	>16	>16	>4	≤0.12	≤1	>1	>8	>8	≤1	16	2	2	>4	>16	>16	>8	>8	>16	>128	64	≤2	>64
21-0005	NR	+	-	-	-	+	32	>16	>16	>16	>4	>2	>4	>1	>8	>8	≤1	16	8	2	>4	>16	>16	>8	>8	>16	>128	64	4	>64
21-0006	NR	+	-	-	-	+	32	>16	>16	>16	>4	>2	>4	>1	>8	>8	≤1	>16	8	2	>4	>16	>16	>8	>8	>16	≤16	64	8	>64
21-0007	Class B	+	-	-	-	+	>32	>16	>16	>16	>4	>2	>4	>1	>8	>8	≤1	>32	8	2	>4	>16	>16	>8	>8	>16	≤16	64	4	>64
21-0008	Class B	+	-	-	-	-	16	>16	>16	>16	>4	2	≤1	>1	>8	>8	2	>32	>16	2	≤1	>16	>16	>8	>8	>16	≤16	≤32	4	>64
21-0009	NR	+	-	-	-	+	32	>16	>16	>16	>4	>2	>4	>1	>8	>8	≤1	16	8	2	>4	>16	>16	>8	>8	>16	≤16	64	4	>64
21-0010	Class B	+	-	-	-	+	32	>16	>16	>16	>4	>2	≤1	>1	>8	>8	>4	>32	16	4	>4	>16	>16	>8	>8	>16	≤16	128	>8	>64
21-0011	Class B	+	-	-	-	+	32	>16	>16	>16	>4	2	≤1	>1	>8	>8	2	>32	>16	2	≤1	>16	>16	>8	>8	>16	32	64	4	>64
21-0012	NR	+	-	-	-	+	32	>16	>16	>16	>4	>2	>4	>1	>8	>8	≤1	>32	8	2	>4	>16	>16	>8	>8	>16	≤16	64	4	>64
21-0013	Class B	+	-	-	-	+	32	>16	>16	>16	>4	>2	≤1	>1	>8	>8	>4	>32	>16	4	>4	>16	>16	>8	>8	>16	≤16	128	>8	>64
21-0014	Class B	+	-	-	-	+	32	>16	>16	>16	>4	2	≤1	>1	>8	>8	2	>32	>16	2	≤1	>16	>16	>8	>8	>16	64	64	4	>64
21-0015	NR	+	-	-	-	+	32	>16	>16	>16	>4	0.25	≤1	>1	>8	>8	≤1	8	2	2	>4	>16	>16	>8	>8	>16	≤16	64	≤2	>64
21-0016	NR	+	-	-	-	+	32	>16	>16	>16	>4	>2	>4	>1	>8	>8	>4	>32	16	4	>4	>16	>16	>8	>8	>16	≤16	128	>8	>64
21-0017	NR	+	-	-	-	+	32	>16	>16	>16	>4	>2	>4	>1	>8	>8	≤1	>32	8	4	>4	>16	>16	>8	>8	>16	>128	>128	8	>64
21-0019	Class B	+	-	-	-	+	32	>16	>16	>16	>4	>2	≤1	>1	>8	>8	>4	>32	>16	>4	>4	>16	>16	>8	>8	>16	≤16	128	>8	>64
21-0020	Class B	+	-	-	-	+	16	>16	>16	>16	>4	2	≤1	>1	>8	>8	2	>32	>16	4	≤1	>16	>16	>8	>8	>16	≤16	≤32	4	>64
21-0021	NR	+	-	-	-	+	8	>16	>16	>16	>4	>2	≤1	>1	8	>8	>4	16	>16	4	≤1	>16	>16	≤0.25	>8	>16	≤16	≤32	>8	>64
21-0023	Class B	+	-	-	-	+	>32	>16	>16	>16	>4	>2	>4	>1	>8	>8	≤1	32	16	4	>4	>16	>16	>8	>8	>16	≤16	128	>8	>64
21-0025	NR	+	-	-	-	+	32	>16	>16	>16	>4	2	>4	>1	>8	>8	≤1	>32	8	2	>4	>16	>16	>8	>8	>16	≤16	64	4	>64
21-0028	Class B	+	-	-	-	+	>32	>16	>16	>16	>4	2	>4	>1	>8	>8	≤1	16	8	2	>4	>16	>16	>8	>8	>16	≤16	64	4	>64
21-0030	Class B	+	-	-	-	+	>32	>16	>16	>16	>4	2	>4	>1	>8	>8	≤1	>16	8	2	>4	>16	>16	>8	>8	>16	≤16	64	4	>64
21-0033	NR	+	-	-	-	+	32	>16	>16	>16	>4	>2	>4	>1	>8	>8	>4	16	>16	>4	>4	>16	>16	>8	>8	>16	≤16	128	>8	>64
21-0034	NR	+	-	-	-	-	16	>16	>16	>16	>4	1	≤1	>1	>8	>8	2	>32	>16	2	≤1	>16	>16	>8	>8	>16	≤16	128	4	>64
21-0035	Class B	+	-	-	-	+	8	>16	>16	>16	>4	>2	≤1	>1	>8	>8	2	>32	>16	2	>4	>16	>16	>8	>8	>16	≤16	≤32	>8	>64
21-0036	Class B	+	-	-	-	+	>32	>16	>16	>16	>4	>2	>4	>1	>8	>8	>4	32	>16	>4	>4	>16	>16	>8	>8	>16	≤16	>128	>8	>64
21-0038	Class B	+	-	-	-	+	8	>16	>16	>16	>4	1	≤1	>1	>8	>8	≤1	>32	>16	2	>4	>16	>16	>8	>8	>16	≤16	64	≤2	>64
21-0042	Class B	+	-	-	-	+	8	>16	>16	>16	>4	1	≤1	>1	>8	>8	≤1	>32	>16	2	>4	>16	>16	>8	>8	>16	≤16	64	≤2	>64
21-0049	NR	+	-	-	-	-	8	>16	>16	>16	>4	>2	≤1	>1	8	>8	>4	>32	>16	4	≤1	>16	>16	>8	>8	>16	≤16	128	>8	>64
21-0050	NR	+	-	-	-	+	>32	>16	>16	>16	>4	2	>4	>1	>8	>8	≤1	16	8	2	>4	>16	>16	>8	>8	>16	≤16	128	8	>64
21-0052	NR	+	-	-	-	+	32	>16	>16	>16	>4	>2	≤1	>1	8	>8	>4	>32	8	4	>4	>16	>16	>8	>8	>16	≤16	128	>8	>64
21-0107	NR *	+	-	-	-	+	8	>16	16	>16	>4	>2	≤1	>1	>8	1	≤1	≤0.5	4	2	>4	>16	>16	0.5	>8	>16	64	>128	>8	>64
21-0113	NR	+	-	-	-	-	≤8	>16	>16	>16	>4	>2	≤1	>1	>8	>8	>4	>16	8	4	>4	>16	>16	>8	>8	>16	≤16	64	>8	>64
21-0114	Class B	+	-	+	-	+	≤8	>16	>16	>16	>4	2	≤1	>1	>8	>8	≤1	>16	≤1	≤1	>4	>16	>16	>8	>8	>16	≤16	≤32	4	>64
21-0144	Class B	+	-	+	-	+	8	>16	>16	>16	>4	>2	≤1	>1	>8	>8	>4	>32	>16	4	≤1	>16	>16	>8	>8	>16	≤16	>128	>8	>64
21-0145	Class B	+	-	-	-	+	>32	>16	>16	>16	>4	>2	≤1	>1	>8	>8	>4	>32	>16	>4	>4	>16	>16	>8	>8	>16	≤16	128	>8	>64
21-0146	Class B	+	-	-	-	+	32	>16	>16	>16	>4	>2	≤1	>1	>8	>8	>4	>32	>16	>4	>4	>16	>16	>8	>8	>16	≤16	128	>8	>64
21-0149	Class B	+	-	-	-	+	>32	>16	>16	>16	>4	>2	≤1	>1	>8	>8	>4	32	>16	4	>4	>16	>16	>8	>8	>16	≤16	128	>8	>64
21-0150	Class B	+	-	-	-	+	32	>16	>16	>16	>4	>2	≤1	>1	>8	>8	>4	32	>16	>4	>4	>16	>16	>8	>8	>16	≤16	128	>8	>64
21-0151	Class B	+	-	-	-	+	32	>16	>16	>16	>4	>2	≤1	>1	>8	>8	>4	>32	>16	>4	>4	>16	>16	>8	>8	>16	≤16	128	>8	>64
21-0152	Class B	+	-	-	-	+	32	>16	>16	>16	>4	>2	≤1	>1	>8	>8	>4	>32	>16	4	>4	>16	>16	>8	>8	>16	≤16	128	>8	>64
21-0154	Class B	+	-	-	-	+	32	>16	>16	>16	>4	>2	≤1	>1	>8	>8	>4	>32	>16	>4	>4	>16	>16	>8	>8	>16	≤16	128	>8	>64
21-0155	NR	+	-	-	-	-	8	>16	>16	>16	>4	>2	≤1	>1	8	>8	>4	>32	>16	4	≤1	>16	>16	>8	>8	>16	≤16	>128	>8	>64
21-0156	Class B	+	-	-	-	-	>32	>16	>16	>16	>4	≤0.12	>4	>1	>8	>8	≤1	32	4	2	>4	>16	≤1	>8	>8	>16	≤16	≤32	≤2	>64
21-0159	Class B	+	-	-	-	-	8	>16	>16	>16	>4	>2	>4	>1	>8	>8	>4	>32	>16	4	≤1	>16	>16	>8	>8	>16	≤16	>128	>8	>64
21-0160	Class B	+	-	-	-	+	32	>16	>16	>16	>4	>2	≤1	>1	>8	>8	>4	>32	>16	4	>4	>16	>16	>8	>8	>16	≤16	128	>8	>64
21-0162	Class B	+	-	-	-	+	8	>16	>16	>16	>4	>2	≤1	>1	>8	>8	2	>32	>16	2	2	>16	>16	>8	>8	>16	≤16	≤32	>8	>64
21-0163	Class B	+	-	-	-	-	16	>16	>16	>16	>4	1	≤1	>1	>8	>8	2	>32	>16	2	≤1	>16	>16	>8	>8	>16	≤16	128	4	>64
21-0164	Class B	+	-	-	-	+	>32	>16	>16	>16	>4	>2	≤1	>1	>8	>8	>4	>32	>16	4	>4	>16	>16	>8	>8	>16	32	>128	>8	>64
21-0165	NR	+	-	-	-	+	32	>16	>16	>16	>4	>2	≤1	>1	>8	>8	>4	>32	>16	4	>4	>16	>16	>8	>8	>16	≤16	128	>8	>64
21-0166	NR	+	-	-	-	+	8	>16	>16	>16	>4	>2	≤1	>1	>8	>8	2	>32	>16	4	2	>16	>16	>8	>8	>16	≤16	≤32	>8	>64
21-0179	Class B	+	-	-	-	+	>32	>16	>16	>16	>4	>2	≤1	>1	>8	>8	>4	>32	4	2	>4	>16	>16	>8	>8	>16	≤16	128	>8	>64
21-0188	Class A	-	+	-	-	-	≤8	>16	16	>16	>4	≤0.12	≤1	>1	≤2	>8	≤1	16	4	2	≤1	>16	>16	4	>8	>16	32	≤32	≤2	>64
21-0189	Class D	-	-	-	-	-	≤8	>16	>16	>16	>4	>2	≤1	>1	≤2	>8	>4	>32	>16	4	>4	>16	>16	2	>8	>16	64	>128	>8	>64
21-0190	Class A	-	+	-	-	-	≤8	>16	16	>16	>4	≤0.12	≤1	>1	≤2	>8	≤1	16	4	2	≤1	>16	>16	4	>8	>16	32	≤32	≤2	>64
21-0218	NR	-	+	-	-	-	≤8	>16	16	>16	>4	2	≤1	>1	>8	>8	≤1	>32	8	2	>4	>16	>16	4	>8	>16	≤16	64	4	>64
21-0223	NR	-	+	-	-	+	>32	>16	>16	>16	>4	>2	≤1	>1	>8	8	>4	8	>16	4	>4	>16	>16	4	>8	>16	≤16	64	>8	>64
21-0224	Class A	+	+	-	-	+	32	>16	>16	>16	>4	>2	≤1	>1	>8	8	>4	8	>16	2	>4	>16	>16	4	>8	>16	≤16	64	>8	>64
21-0225	Class A	+	+	-	-	+	>32	>16	>16	>16	>4	>2	≤1	>1	>8	8	>4	8	>16	2	>4	>16	>16	4	>8	>16	≤16	64	>8	>64
21-0227	Class A	-	+	-	+	+	32	>16	>16	>16	>4	>2	≤1	>1	>8	8	>4	8	>16	2	>4	>16	>16	4	>8	>16	≤16	64	>8	>64
21-0228	NR	+	-	-	-	+	32	>16	>16	>16	>4	>2	≤1	>1	>8	4	>4	4	>16	2	>4	>16	>16	1	>8	>16	≤16	64	>8	>64
21-0229	Class A	-	+	-	-	+	>32	>16	>16	>16	>4	>2	≤1	>1	>8	8	>4	8	>16	2	>4	>16	>16	2	>8	>16	≤16	64	>8	>64
21-0230	Class A	+	+	-	-	+	32	>16	>16	>16	>4	>2	≤1	>1	>8	8	>4	8	>16	2	>4	>16	>16	4	>8	>16	≤16	64	>8	>64
21-0231	Class A	-	+	-	-	-	32	>16	16	>16	>4	≤0.12	≤1	>1	≤2	>8	≤1	16	>16	≤1	>4	>16	>16	2	>8	>16	≤16	≤32	≤2	>64
21-0232	Class A	-	+	-	-	+	32	>16	>16	>16	>4	>2	≤1	>1	>8	8	>4	8	>16	2	>4	>16	>16	4	>8	>16	≤16	64	>8	>64
21-0234	Class A	-	+	-	-	+	32	>16	>16	>16	>4	>2	≤1	>1	>8	8	>4	8	>16	2	>4	>16	>16	2	>8	>16	≤16	64	>8	>64
21-0235	Class A	+	+	-	-	-	>32	>16	>16	>16	>4	>2	≤1	>1	>8	>8	>4	16	>16	2	>4	>16	>16	4	>8	>16	≤16	64	>8	>64
21-0237	NR	-	+	+	-	+	32	>16	>16	>16	>4	>2	≤1	>1	>8	>8	>4	16	>16	2	>4	>16	>16	2	>8	>16	≤16	64	>8	>64
21-0239	NR	+	-	-	-	+	32	>16	>16	>16	>4	>2	≤1	>1	>8	8	>4	8	>16	4	>4	>16	>16	4	>8	>16	≤16	64	>8	>64
21-0242	NR	-	-	+	-	+	>32	>16	>16	>16	>4	>2	≤1	>1	>8	8	>4	16	>16	4	>4	>16	>16	4	>8	>16	≤16	64	>8	>64
21-0245	Class B	+	-	-	-	+	>32	>16	>16	>16	>4	>2	>4	>1	>8	>8	>4	>32	>16	>4	>4	>16	>16	>8	>8	>16	>128	128	>8	>64
21-0306	NR	+	-	-	-	+	>32	>16	>16	>16	>4	>2	>4	>1	8	>8	>4	>32	>16	4	>4	>16	>16	>8	>8	>16	>128	128	>8	>64
21-0307	Class B	+	-	-	-	-	32	>16	>16	>16	>4	>2	>4	>1	≤2	>8	>4	>32	>16	4	>4	>16	>16	>8	>8	>16	≤16	≤32	>8	>64

21-0308	NR	+	-	-	-	+	32	>16	>16	>16	>4	>2	>4	>1	≤2	>8	>4	>32	>16	4	>4	>16	>16	>8	>8	>16	≤16	128	>8	>64
21-0337	NR	+	-	+	-	+	32	>16	>16	>16	>4	>2	≤1	>1	4	>8	>4	>32	8	2	>4	>16	>16	>8	>8	>16	≤16	64	>8	>64
21-0596	Class B	+	-	-	-	-	8	>16	>16	>16	>4	>2	≤1	>1	>8	>8	>4	>32	>16	>4	≤1	>16	>16	>8	>8	>16	≤16	128	>8	>64
21-0597	Class B	+	-	-	-	-	16	>16	>16	>16	>4	>2	≤1	>1	>8	>8	>4	>32	>16	4	>4	>16	>16	>8	>8	>16	≤16	>128	>8	>64
21-0603	Class B	+	-	-	-	+	32	>16	>16	>16	>4	>2	≤1	>1	>8	>8	>4	>32	>16	4	>4	>16	>16	>8	>8	>16	≤16	128	>8	>64
21-0607	Class B	+	-	-	-	-	>32	>16	>16	>16	>4	>2	≤1	>1	>8	>8	>4	>32	>16	4	>4	>16	>16	>8	>8	>16	≤16	128	>8	>64
21-0608	Class B	+	-	-	-	+	8	>16	>16	>16	>4	>2	≤1	>1	>8	>8	2	>32	>16	2	2	>16	>16	>8	>8	>16	≤16	≤32	>8	>64
21-0609	NR	+	-	-	-	+	8	>16	>16	>16	>4	>2	≤1	>1	>8	>8	2	>32	>16	4	2	>16	>16	>8	>8	>16	≤16	≤32	>8	>64
21-0610	NR	+	-	+	-	+	8	>16	>16	>16	>4	>2	≤1	>1	4	>8	2	>32	>16	4	>4	>16	>16	>8	>8	>16	≤16	≤32	>8	>64
21-0633	Class B	+	-	-	-	-	≤8	>16	>16	>16	>4	1	≤1	>1	>8	>8	≤1	>32	≤1	≤1	>4	>16	>16	>8	>8	>16	≤16	≤32	4	>64
21-0801	Class B	+	-	-	-	-	≤8	>16	>16	>16	>4	1	≤1	>1	≤2	>8	≤1	>32	4	2	>4	>16	8	>8	>8	>16	≤16	≤32	4	>64
21-0802	NR	-	+	-	-	+	>32	>16	>16	>16	>4	>2	≤1	>1	4	>8	>4	>32	4	4	>4	>16	>16	2	>8	>16	≤16	>128	>8	>64
21-1015	Class B	+	-	-	-	-	>32	>16	>16	>16	>4	>2	≤1	>1	≤2	>8	>4	16	4	2	>4	>16	>16	>8	>8	>16	≤16	64	>8	>64
21-1021	Class B	+	-	+	-	-	32	>16	>16	>16	>4	>2	≤1	>1	>8	>8	>4	>32	>16	4	>4	>16	>16	>8	>8	>16	32	128	>8	>64
21-1024	Class B	+	-	-	-	-	32	>16	>16	>16	>4	>2	≤1	>1	>8	>8	>4	>32	16	4	>4	>16	>16	>8	>8	>16	≤16	128	>8	>64
21-1028	Class B	+	-	-	-	-	8	>16	>16	>16	>4	>2	≤1	>1	>8	>8	>4	>32	>16	>4	>4	>16	>16	>8	>8	>16	≤16	64	>8	>64
21-1032	CP	+	-	+	-	-	8	>16	>16	>16	>4	2	≤1	>1	>8	>8	2	>32	>16	2	≤1	>16	>16	>8	>8	>16	32	64	4	>64
21-1074	NR	+	-	-	-	-	>32	>16	>16	>16	>4	>2	≤1	>1	>8	>8	≤1	>32	8	4	>4	>16	>16	>8	>8	>16	≤16	128	≤2	>64
21-1097	NR	+	-	-	-	-	32	>16	>16	>16	>4	>2	≤1	>1	>8	>8	>4	>32	16	4	>4	>16	>16	>8	>8	>16	≤16	128	>8	>64
21-1098	NR	+	-	-	-	-	16	>16	>16	>16	>4	>2	≤1	>1	>8	>8	>4	>32	8	4	>4	>16	>16	>8	>8	>16	≤16	>128	>8	>64
21-1099	NR	-	-	+	-	-	32	>16		>16	>4	0.25	≤1	>1	>8	>8		16	>8	2	>4	>16		>8						>64
21-1099	Class B	-	-	+	-	-	32	>16	>16	>16	>4	0.25	≤1	>1	>8	>8	≤1	32	>16	2	>4	>16	>16	>8	>8	>16	≤16	≤32	≤2	>64
21-1100	NR	+	-	-	-	-	32	>16	>16	>16	>4	>2	≤1	>1	>8	>8	>4	>32	>16	4	>4	>16	>16	>8	>8	>16	32	128	>8	>64
21-1104	NR	-	-	-	-	-	32	>16	>16	>16	>4	>2	≤1	>1	>8	>8	>4	>32	16	4	>4	>16	>16	>8	>8	>16	≤16	128	>8	>64
21-1105	Class B	-	-	-	-	-	>32	>16	>16	>16	>4	>2	≤1	>1	>8	>8	>4	>32	16	4	>4	>16	>16	>8	>8	>16	≤16	>128	>8	>64
21-1106	NR	+	-	-	-	-	32	>16	>16	>16	>4	>2	≤1	>1	4	>8	>4	>32	16	4	>4	>16	>16	>8	>8	>16	≤16	128	>8	>64
21-1107	NR	-	-	-	-	-	32	>16	>16	>16	>4	>2	≤1	>1	4	>8	>4	>32	16	4	>4	>16	>16	>8	>8	>16	≤16	128	>8	>64
21-1108	NR	+	-	-	-	-	>32	>16	>16	>16	>4	>2	>4	>1	8	>8	>4	2	8	4	>4	>16	>16	>8	>8	>16	>128	64	>8	>64
21-1111	NR	-	-	-	-	-	32	>16	>16	>16	>4	>2	>4	>1	>8	>8	>4	>32	8	4	>4	>16	>16	>8	>8	>16	64	128	>8	>64
21-1114	Class B	+	-	-	-	-	>32	>16	>16	>16	>4	>2	>4	>1	>8	>8	>4	>32	>16	4	>4	>16	>16	>8	>8	>16	128	>128	>8	>64
21-1118	Class B	+	-	-	-	-	32	>16	>16	>16	>4	>2	≤1	>1	>8	>8	>4	>32	>16	4	>4	>16	>16	>8	>8	>16	32	128	>8	>64
21-1119	NR	+	-	-	-	-	32	>16	>16	>16	>4	>2	≤1	>1	>8	>8	>4	>32	16	4	>4	>16	>16	>8	>8	>16	≤16	128	>8	>64
21-1120	NR	-	+	+	-	-	≤8	8	8	8	>4	≤0.12	>4	>1	≤2	8	≤1	4	4	2	≤1	>16	>16	1	8	>16	≤16	128	≤2	>64
21-1123	Class B	-	-	-	-	-	≤8	>16	>16	>16	>4	>2	≤1	>1	>8	>8	>4	>32	>16	>4	>4	>16	>16	>8	>8	>16	128	>128	>8	>64
21-1124	NR	-	-	-	-	-	≤8	≤1	≤4	≤2	2	≤0.12	≤1	>1	≤2	2	≤1	4	2	2	≤1	>16	>16	0.5	4	>16	≤16	128	≤2	>64
21-1125	Class B	+	-	-	-	-	≤8	>16	>16	>16	>4	>2	≤1	>1	>8	>8	>4	>32	>16	4	>4	>16	>16	>8	>8	>16	≤16	128	>8	>64
21-1126	NR	-	-	-	-	-	32	>16	>16	>16	>4	>2	>4	>1	>8	>8	≤1	32	8	4	>4	>16	>16	>8	>8	>16	32	128	8	>64
21-1129	NR	+	-	-	-	-	32	>16	>16	>16	>4	2	>4	>1	>8	>8	≤1	32	8	2	>4	>16	>16	>8	>8	>16	>128	128	8	>64
22-1272	Class B	+	-	-	-	-	≤8	>16	>16	>16	>4	>2	>4	>1	≤2	>8	>4	>32	>16	4	>4	>16	≤1	>8	>8	>16	>128	>128	>8	>64
22-1289	NR	+	-	-	-	-	>32	>16	>16	>16	>4	>2	≤1	>1	>8	>8	>4	>32	>16	2	>4	>16	>16	8	>8	>16	≤16	>128	>8	>64
22-1302	Class B	+	-	-	-	-	>32	>16	>16	>16	>4	2	≤1	>1	8	>8	≤1	>32	2	2	≤1	>16	16	>8	>8	>16	≤16	≤32	4	>64
22-1303	NR	+	-	-	-	-	32	>16	>16	>16	>4	2	≤1	>1	4	>8	≤1	>32	4	2	≤1	>16	>16	>8	>8	>16	≤16	64	8	>64
22-1304	Class B	+	-	-	-	-	32	>16	>16	>16	>4	≤0.12	≤1	>1	≤2	4	≤1	8	4	2	>4	>16	≤1	>8	>8	>16	≤16	128	≤2	>64
22-1305	Class B	+	-	-	-	-	16	>16	>16	>16	>4	>2	>4	>1	8	>8	>4	>32	>16	4	>4	>16	>16	>8	>8	>16	64	128	>8	>64
22-1312	NR	+	-	-	-	-	32	>16	>16	>16	>4	1	≤1	>1	≤2	>8	≤1	>32	8	2	>4	>16	>16	>8	>8	>16	32	64	4	>64
22-1315	NR *	-	-	-	-	-	≤8	>16	>16	>16	>4	>2	≤1	>1	>8	4	≤1	8	4	2	>4	>16	>16	4	>8	>16	32	128	>8	>64
22-1317	NR	+	-	-	-	-	32	>16	>16	>16	>4	1	≤1	>1	≤2	>8	≤1	>32	4	4	>4	>16	>16	>8	>8	>16	64	64	≤2	>64
22-1320	Class A	-	+	-	-	-	8	>16	16	>16	>4	2	≤1	>1	>8	>8	≤1	32	4	2	>4	>16	>16	2	>8	>16	≤16	≤32	4	>64
22-1322	Class A	-	+	-	-	-	8	>16	16	>16	>4	2	≤1	>1	>8	>8	≤1	16	4	2	>4	>16	>16	2	>8	>16	≤16	≤32	4	>64
22-1323	Class B	+	-	-	-	-	32	>16	>16	>16	>4	>2	>4	>1	>8	>8	>4	>32	>16	4	>4	>16	>16	>8	>8	>16	≤16	128	>8	>64
22-1327	NR	+	-	-	-	-	>32	>16	>16	>16	>4	>2	≤1	>1	8	>8	>4	>32	>16	4	>4	>16	>16	>8	>8	>16	>128	128	>8	>64
22-1328	NR	-	-	-	-	-	≤8	>16	>16	>16	>4	>2	>4	>1	>8	1	2	4	8	2	>4	>16	>16	1	2	>16	≤16	128	>8	32
22-1329	Class A	-	+	-	-	-	8	>16	16	>16	>4	2	≤1	>1	>8	4	≤1	4	2	2	>4	>16	>16	≤0.25	>8	>16	≤16	≤32	4	>64
22-1383	NR	-	-	-	-	-	≤8	>16	≤4	>16	>4	>2	≤1	>1	>8	>8	≤1	>16	4	≤1	>4	>16	≤1	>8	≤1	≤4	≤16	≤32	≤2	>64
22-1423	Class B	+	-	-	-	-	>32	>16	>16	>16	>4	>2	≤1	>1	>8	>8	>4	>32	>16	2	>4	>16	>16	>8	>8	>16	≤16	>128	>8	>64
22-1429	NR	-	-	-	-	-	≤8	>16	≤4	>16	>4	2	≤1	>1	>8	≤0.25	≤1	4	>16	2	>4	>16	>16	>8	>8	>16	≤16	128	4	64
22-1705	NR *	-	-	-	-	-	≤8	>16	>16	>16	>4	>2	>4	>1	>8	2	2	8	8	2	>4	>16	>16	1	4	>16	≤16	128	>8	64
22-1713	Class B	-	+	-	-	-	≤8	>16	>16	>16	>4	>2	≤1	>1	>8	≤0.25	>4	>32	>16	>4	>4	>16	>16	>8	>8	>16	128	>128	>8	>64
22-1738	Class B	+	-	-	-	-	>32	>16	>16	>16	>4	>2	>4	>1	>8	>8	>4	>32	16	4	>4	>16	>16	>8	>8	>16	≤16	128	>8	>64
22-1766	Class A	-	+	-	-	-	≤8	>16	16	>16	>4	>2	≤1	>1	>8	>8	>4	32	4	≤1	>4	>16	>16	8	>8	>16	≤16	128	>8	>64
22-1767	Class A	-	+	-	-	-	≤8	>16	16	>16	>4	>2	≤1	>1	>8	>8	>4	16	4	2	>4	>16	>16	4	>8	>16	≤16	128	>8	>64
22-1768	Class A	-	+	-	-	-	≤8	>16	16	>16	>4	>2	≤1	>1	>8	>8	>4	>16	4	2	>4	>16	>16	4	>8	>16	64	128	>8	>64
22-1786	Class B	+	-	-	-	-	≤8	>16	>16	>16	>4	>2	≤1	>1	>8	>8	2	>16	4	2	>4	>16	>16	>8	>8	>16	≤16	≤32	8	>64
22-1811	Class B	+	-	-	-	-	>32	>16	>16	>16	>4	>2	≤1	>1	>8	>8	>4	32	>16	4	>4	>16	>16	>8	>8	>16	≤16	>128	>8	>64
22-1820	NR	-	-	-	-	-	≤8	>16	>16	>16	>4	>2	≤1	>1	>8	>8	>4	>32	16	4	>4	>16	>16	8	>8	>16	64	>128	>8	>64
22-1867	CP	-	-	-	-	-	8	>16	>16	>16	>4	>2	≤1	>1	>8	2	>4	8	8	2	>4	>16	>16	2	>8	>16	64	>128	>8	>64
22-1873	NR	-	-	+	-	-	32	>16	>16	>16	>4	>2	≤1	1	>8	8	>4	4	4	2	>4	>16	>16	1	>8	>16	≤16	64	>8	>64
22-1883	Class B	+	-	-	-	-	>32	>16	>16	>16	>4	>2	>4	>1	>8	>8	>4	>32	8	2	>4	>16	>16	>8	>8	>16	32	128	>8	>64
22-1905	Class B	+	-	-	-	-	32	>16	>16	>16	>4	>2	>4	>1	>8	>8	>4	>32	>16	4	>4	>16	>16	>8	>8	>16	≤16	128	>8	>64
22-1995	Class B	-	-	-	-	-	≤8	>16	>16	>16	>4	>2	≤1	>1	≤2	2	≤1	8	>16	>4	>4	>16	>16	2	>8	>16	64	>128	8	>64
22-2174	Class B	+	-	-	-	-	32	>16	>16	>16	>4	>2	>4	>1	>8	>8	>4	>32	16	4	>4	>16	>16	>8	>8	>16	≤16	128	>8	>64
22-2176	Class D	-	-	-	-	+	>32	>16	>16	>16	>4	>2	≤1	>1	>8	>8	>4	16	16	4	>4	>16	>16	>8	>8	>16	≤16	128	>8	>64
22-2178	Class B	+	-	-	-	-	>32	>16	>16	>16	>4	>2	≤1	>1	>8	>8	>4	>32	>16	4	>4	>16	>16	>8	>8	>16	≤16	64	>8	>64
22-2187	Class B	+	-	-	-	-	>32	16	>16	>16	>4	≤0.12	≤1	>1	≤2	>8	≤1	16	4	2	>4	>16	≤1	>8	>8	>16	≤16	≤32	≤2	>64
22-2202	Class B	+	-	-	-	-	>32	16	>16	>16	>4	≤0.12	≤1	>1	≤2	>8	≤1	8	4	2	>4	>16	≤1	>8	>8	>16	≤16	≤32	≤2	>64
23-2142	NR	-	-	-	+	-	≤8	>16	>16	>16	>4	>2	≤1	>1	>8	>8	>4	>32	4	2	>2	>16	>16	>8	>8	>16	32	128	>8	>64
23-2205	Class B	-	-	-	-	-	>32	16	>16	>16	>4	≤0.12	≤1	>1	≤2	>8	≤1	16	4	2	>4	>16	≤1	>8	>8	>16	≤16	≤32	≤2	>64
23-2219	Class B	+	-	-	-	-	>32	>16	>16	>16	>4	≤0.12	≤1	>1	≤2	>8	≤1	8	4	2	>4	>16	≤1	>8	>8	>16	≤16	≤32	≤2	>64

AMK: amikacin, AMP: ampicillin, FEP: cefepime, FOX, cefoxitin, CAZ: ceftazidime, CRO: ceftriaxone, CIP: ciprofloxacin, COL: colistin, ETP: ertapenem, GEN: gentamicin, IPM: imipenem, LVX: levofloxacin, MEM: meropenem, MNO: minocycline, TGC: tigecycline, SXT: trimethoprim/sulfamethoxazole, SAM: ampicillin/sulbactam, ATM: aztreonam, CZA: ceftazidime-avibactam, CZT: ceftolozane/tazobactam, CXM: cefuroxime, FOS: fosfomycin, NIT: nitrofurantoin, NOR: norfloxacin, TZP: piperacillin/tazobactam. NR: no recommendation, CP: carbapenemase producer. * Extended spectrum β–lactamase producer. – negative, + positive

Supplementary Table S4. CPO report, carbapenemase encoding genes and minimal inhibitory concentration (μg/ml) in *P. aeruginosa* clinical isolates

ID	CPO report	*bla*VIM	*bla*IMP	*bla*GES	AMK	FEP	CAZ	CIP	COL	GEN	IPM	LVX	MEM	TGC	ATM	CZA	CZT	FOS	NIT	NOR	TZP
21-0075	Class B	+	-	-	>32	>16	>16	>2	≤1	8	>8	>4	>32	>4	8	>8	>8	32	>128	>8	64
21-0077	Class B	+	-	-	>32	>16	>16	>2	≤1	8	>8	>4	>32	>4	8	>8	>8	≤16	>128	>8	64
21-0078	Class B	+	-	-	>32	>16	>16	>2	>4	>8	>8	>4	>32	>4	16	>8	>8	64	>128	>8	>64
21-0680	CP	-	+	+	>32	>16	>16	>2	≤1	>8	>8	>4	>32	>4	>16	>8	>8	>64	>128	>8	>64
21-700	NR	-	-	-	≤4	4	4	2	≤1	4	4	4	≤0.5	>4	2	1	≤1	>64	>128	4	32
20-0490	NR	-	-	-	≤8	8	8	1	≤1	≤2	>8	4	32	>4	>16	8	≤1	64	>128	4	16
20-0503	CP	-	-	+	>32	>16	>16	>2	≤1	>8	>8	>4	>32	>4	>16	>8	>8	>64	>128	>8	>64
21-0063	CP	-	-	+	>32	>16	>16	>2	≤1	>8	>8	>4	>32	>4	>16	>8	>8	>64	>128	>8	>64
21-0069	Class B	+	-	-	>32	>16	>16	>2	2	>8	>8	>4	>32	>4	8	>8	>8	32	>128	>8	64
21-0088	CP	+	-	-	16	>16	>16	>2	≤1	≤2	>8	>4	>32	>4	>16	>8	>8	≤16	>128	>8	64
21-0089	Class A	-	-	+	>32	>16	>16	>2	≤1	>8	>8	>4	>32	>4	>16	>8	>8	32	>128	>8	>64
21-0220	NR	-	-	-	≤8	8	8	0.5	≤1	≤2	>8	2	16	>4	>16	8	≤1	64	>128	≤2	16
21-0222	Class B	+	-	-	32	>16	>16	>2	≤1	4	>8	>4	>32	>4	>16	>8	>8	≤16	>128	>8	64
21-0249	NR	-	-	-	8	>16	>16	>2	≤1	4	>8	>4	>32	4	>16	>8	>8	>64	>128	>8	>64
21-0255	CP	-	-	-	16	16	>16	>2	≤1	>8	>8	>4	>32	>4	>16	>8	4	64	>128	>8	64
21-0257	CP	+	-	-	≤8	8	>16	>2	≤1	>8	>8	>4	>32	>4	>16	>8	8	64	>128	>8	32
21-0258	Class B	+	-	-	32	>16	>16	>2	≤1	4	>8	>4	>32	>4	>16	>8	>8	≤16	>128	>8	64
21-0259	CP	+	-	-	32	8	>16	>2	≤1	>8	>8	>4	>32	>4	>16	>8	4	64	>128	>8	32
21-0262	CP	+	-	-	32	>16	>16	>2	≤1	4	>8	>4	>32	>4	16	>8	>8	≤16	>128	>8	64
21-0263	Class B	+	-	-	32	>16	>16	>2	≤1	4	>8	>4	>32	>4	>16	>8	>8	≤16	>128	>8	64
21-0264	CP	-	-	-	32	8	16	>2	≤1	>8	>8	>4	32	>4	8	4	≤1	32	>128	>8	8
21-0269	Class A	+	-	-	≤8	8	8	0.5	≤1	≤2	>8	2	16	>4	>16	8	≤1	64	>128	≤2	16
21-0270	Class B	+	-	-	32	>16	>16	>2	≤1	≤2	>8	>4	>32	>4	>16	>8	>8	>64	>128	>8	64
21-0280	NR	-	-	-	32	>16	>16	2	≤1	≤2	4	>4	4	>4	8	4	>8	>64	>128	8	32
21-0282	NR	-	-	-	≤8	8	4	2	≤1	≤2	>8	>4	4	>4	8	4	≤1	>64	>128	8	8
21-0283	NR	-	-	-	32	8	8	>2	>4	≤2	>8	>4	16	>4	>16	4	≤1	>64	>128	>8	8
21-0296	Class D	-	-	-	>32	>16	>16	>2	≤1	>8	>8	>4	>32	>4	>16	>8	>8	32	>128	>8	>64
21-0317	Class B	-	-	-	>32	>16	>16	>2	≤1	>8	>8	>4	>32	>4	>16	>8	>8	>64	>128	>8	32
21-0318	NR	-	-	-	>32	>16	>16	>2	≤1	>8	>8	>4	>32	>4	≤1	>8	>8	>64	>128	>8	64
21-0319	CP	-	+	-	>32	>16	>16	>2	≤1	>8	>8	>4	>32	>4	>16	>8	>8	>64	>128	>8	64
21-0320	CP	-	-	+	>32	>16	>16	>2	≤1	>8	>8	>4	>32	>4	>16	>8	>8	>64	>128	>8	>64
21-0321	CP	-	-	-	>32	>16	>16	>2	≤1	>8	>8	>4	>32	>4	>16	>8	>8	>64	>128	>8	64
21-0369	NR	-	-	-	≤8	4	4	≤0.12	≤1	≤2	>8	≤1	8	>4	8	2	≤1	64	>128	≤2	≤4
21-0447	NR	-	-	-	≤8	16	8	>2	≤1	≤2	>8	>4	32	>4	>16	8	≤1	≤16	>128	>8	32
21-0449	CP	-	+	-	8	16	16	1	≤1	4	>8	4	16	>4	>16	8	2	>64	>128	4	16
21-0454	NR	-	-	-	32	16	8	>2	≤1	>8	>8	>4	32	>4	>16	4	2	>64	>128	8	8
21-0486	CP	-	-	-	16	>16	>16	0.5	≤1	8	>8	4	>32	>4	>16	>8	>8	32	>128	≤2	32
21-0510	NR	-	-	-	≤4	8	8	>2	≤1	≤2	>8	>4	16	>4	>16	4	≤1	32	>128	8	16
21-0541	NR	-	-	-	≤4	8	8	0.25	≤1	≤2	>8	2	16	>4	>16	4	≤1	64	>128	≤2	16
21-0543	Class A	-	-	-	>32	>16	>16	>2	≤1	>8	>8	>4	32	>4	>16	8	>8	32	>128	>8	32
21-0549	NR	-	-	-	≤4	2	4	≤0.12	≤1	≤2	>8	≤1	4	>4	8	2	≤1	64	>128	≤2	≤4
21-0554	NR	-	-	-	≤8	4	4	≤0.12	≤1	≤2	>8	≤1	4	>4	8	2	≤1	64	>128	≤2	≤4
21-0575	Class A	-	-	-	≤8	16	>16	0.5	≤1	≤2	>8	2	32	>4	>16	4	≤1	64	>128	≤2	>64
21-0617	Class B	-	+	+	>32	>16	>16	2	≤1	8	>8	>4	16	>4	>16	>8	>8	32	>128	>8	64
21-0618	NR	-	-	-	≤8	8	8	0.25	≤1	≤2	>8	≤1	16	>4	>16	4	≤1	32	>128	≤2	16
21-0629	NR	-	-	-	≤4	4	4	≤0.12	≤1	≤2	>8	≤1	8	>4	8	2	≤1	32	>128	≤2	8
21-0631	NR	-	-	-	≤8	16	>16	>2	≤1	>8	>8		>16	>4		>8					32
21-0631	Class D	-	-	-	≤4	8	>16	>2	≤1	>8	>8	>4	>32	>4	>16	>8	2	64	>128	>8	32
21-0634	CP	-	-	-	≤8	16	8	1	≤1	≤2	>8	2	16	>4	>16	8	≤1	64	>128	≤2	16
21-0635	Class B	+	-	-	>32	>16	>16	>2	≤1	≤2	>8	>4	>32	>4	>16	>8	>8	≤16	>128	>8	64
21-0636	Class B	-	-	-	16	>16	>16	>2	≤1	>8	>8	>4	32	>4	>16	>8	4	32	>128	>8	>64
21-0637	NR	-	-	+	>32	>16	>16	>2	≤1	>8	>8		16	>4		8					32
21-0637	Class D	-	-	+	>32	>16	>16	>2	≤1	>8	>8	>4	16	>4	4	4	>8	32	>128	>8	32
21-0638	NR	-	+	-	16	>16	>16	>2	≤1	≤2	>8		>16	>4		>8					64
21-0638	Class B	-	+	-	16	16	>16	>2	≤1	≤2	>8	>4	>32	>4	>16	>8	>8	≤16	>128	>8	64
21-0647	CP	-	+	+	>32	>16	>16	>2	≤1	>8	>8	>4	>32	>4	>16	>8	>8	>64	>128	>8	>64
21-0648	CP	-	-	+	>32	>16	>16	>2	≤1	>8	>8	>4	>32	>4	>16	>8	>8	>64	>128	>8	>64
21-0650	CP	-	-	+	>32	>16	>16	>2	≤1	>8	>8	>4	>32	>4	>16	>8	>8	>64	>128	>8	>64

21-0659	NR	-	-	-	≤4	16	8	1	≤1	≤2	>8	4	16	>4	>16	8	≤1	64	>128	4	32
21-0660	Class B	-	+	+	>32	>16	>16	>2	≤1	>8	>8	>4	>32	>4	8	>8	>8	32	>128	>8	64
21-0661	Class A	-	-	+	>32	>16	>16	>2	≤1	>8	>8	>4	32	>4	>16	8	>8	32	>128	>8	32
21-0662	NR	-	-	+	>32	>16	>16	>2	≤1	>8	>8		>16	>4		8					32
21-0662	Class A	-	-	+	>32	>16	>16	>2	≤1	>8	>8	>4	32	>4	>16	8	>8	32	>128	>8	64
21-0663	CP	-	-	+	>32	>16	>16	>2	≤1	>8	>8	>4	>32	>4	>16	>8	>8	>64	>128	>8	>64
21-0664	CP	-	-	+	>32	>16	>16	>2	≤1	>8	>8	>4	>32	>4	>16	>8	>8	>64	>128	>8	>64
21-0666	NR	+	-	-	>32	>16	>16	>2	≤1	>8	>8		>16	>4		>8					64
21-0666	Class B	+	-	-	16	4	4	≤0.12	≤1	>8	>8	≤1	8	>4	8	2	≤1	32	>128	≤2	8
21-0675	Class A	-	+	+	>32	>16	>16	>2	≤1	>8	>8	>4	32	>4	>16	8	>8	64	>128	>8	32
21-0679	CP	-	-	+	>32	>16	>16	>2	≤1	>8	>8	>4	>32	>4	>16	>8	>8	>64	>128	>8	>64
21-0785	NR	-	-	-	≤8	8	>16	>2	≤1	≤2	>8	>4	32	>4	16	2	≤1	>64	>128	>8	16
21-0787	Class B	+	-	-	32	16	>16	>2	≤1	4	>8	>4	>32	>4	16	>8	>8	≤16	>128	>8	32
21-0788	Class D	-	-	-	>32	>16	>16	>2	≤1	>8	2	>4	4	>4	>16	>8	>8	32	>128	>8	>64
21-0789	Class B	+	-	-	32	16	16	>2	≤1	≤2	>8	>4	>32	>4	8	>8	>8	≤16	>128	>8	16
21-0790	Class B	+	+	-	32	>16	>16	>2	≤1	≤2	>8	>4	>32	>4	16	>8	>8	≤16	>128	>8	64
21-0805	NR	-	-	-	≤8	4	4	≤0.12	≤1	≤2	>8	≤1	8	>4	8	4	≤1	64	>128	≤2	8
21-0806	Class D	-	-	-	≤8	>16	>16	≤0.12	≤1	≤2	>8	≤1	16	>4	>16	8	2	≤16	>128	≤2	>64
21-0807	Class A	-	-	-	≤8	16	16	1	≤1	≤2	>8	2	32	>4	>16	8	≤1	64	>128	≤2	32
21-0808	NR	-	-	-	≤8	8	8	1	≤1	≤2	>8	4	16	>4	>16	8	≤1		>128	4	32
21-0811	Class A	-	-	-	≤8	16	8	0.25	≤1	≤2	>8	2	32	>4	>16	8	≤1	>64	>128	≤2	16
21-0817	Class A	-	-	-	≤8	16	16	>2	≤1	≤2	>8	>4	32	>4	>16	>8	≤1	64	>128	>8	32
21-0936	NR	-	-	-	≤8	8	4	1	≤1	≤2	>8	2	8	>4	8	2	≤1	64	>128	≤2	8
21-0947	Class D	-	-	+	8	>16	>16	>2	≤1	4	>8	>4	16	>4	>16	>8	8	>64	>128	>8	>64
21-0951	NR	-	-	-	≤8	16	>16	>2	≤1	≤2	>8	>4	8	>4	2	4	2	>64	>128	>8	>64
21-0989	Class A	-	-	+	>32	>16	>16	>2	≤1	>8	>8	>4	>32	>4	>16	>8	>8	>64	>128	>8	>64
21-0990	CP	-	+	-	>32	>16	>16	>2	≤1	>8	>8	>4	>32	>4	>16	>8	>8	>64	>128	>8	64
21-0991	Class A	-	-	+	>32	>16	>16	>2	≤1	>8	>8	>4	>32	>4	>16	>8	>8	>64	>128	>8	>64
21-0993	Class B	+	-	-	>32	>16	>16	>2	2	>8	>8	>4	>32	>4	8	>8	>8	≤16	>128	>8	64
21-0994	Class D	-	-	-	>32	8	8	>2	≤1	>8	>8	>4	16	>4	≤1	4	≤1	>64	>128	>8	8
21-0995	Class B	+	-	-	>32	>16	>16	>2	≤1	>8	>8	>4	>32	>4	4	>8	>8	32	>128	>8	64
21-1037	Class D	-	-	-	>32	>16	>16	>2	≤1	>8	>8	>4	>32	>4	>16	>8	>8	32	>128	>8	>64
21-1038	Class B	+	-	-	>32	>16	>16	>2	≤1	>8	>8	>4	>32	>4	8	>8	>8	≤16	>128	>8	64
21-1041	NR	-	-	-	>32	16	2	0.25	≤1	4	>8	≤1	8	>4	≤1	2	≤1	64	>128	≤2	8
21-1050	CP	-	-	+	>32	>16	>16	>2	≤1	>8	>8	>4	>32	>4	>16	>8	>8	>64	>128	>8	>64
21-1055	CP	-	-	+	>32	>16	>16	>2	≤1	>8	>8	2	>16	>4	>16	>8	≤1	64	>128	≤2	>64
21-1059	CP	+	-	+	>32	>16	>16	>2	≤1	8	>8	>4	>32	>4	16	>8	>8	≤16	>128	>8	64
21-1060	Class B	+	-	-	>32	>16	>16	>2	2	>8	>8	>4	>32	>4	8	>8	>8	32	>128	>8	>64
21-1062	CP	-	-	+	>32	>16	>16	>2	≤1	>8	>8	>4	>32	>4	>16	>8	>8	>64	>128	>8	>64
21-1067	Class D	-	-	+	>32	>16	>16	>2	≤1	>8	>8	>4	>32	>4	>16	>8	>8	32	>128	>8	>64
21-1068	Class D	-	-	+	>32	>16	>16	>2	≤1	>8	4	>4	16	>4	>16	>8	>8	64	>128	>8	>64
21-1077	CP	-	-	-	>32	>16	>16	>2	≤1	>8	>8	>4	>32	>4	>16	>8	>8	>64	>128	>8	>64
21-1079	Class B	+	-	-	>32	>16	>16	>2	≤1	>8	>8	>4	>32	>4	8	>8	>8	32	>128	>8	64
21-1081	Class A	-	-	+	>32	>16	>16	>2	≤1	>8	>8	>4	>32	>4	>16	>8	>8	32	>128	>8	>64
21-1083	Class D	-	-	-	>32	>16	>16	>2	≤1	>8	>8	>4	>32	>4	>16	>8	>8	32	>128	>8	>64
21-1087	CP	-	-	+	>32	>16	>16	>2	≤1	>8	>8	>4	>32	>4	>16	>8	>8	32	>128	>8	>64
21-1136	Class D	-	-	-	>32	>16	>16	>2	≤1	>8	>8	>4	>32	>4	>16	>8	>8	32	>128	>8	>64
21-1134	NR	+	-	-	≤8	>16	>16	>2	≤1	≤2	>8	≤1	16	>4	2	4	2	≤16	>128	≤2	64
21-1142	CP	-	-	+	>32	>16	>16	>2	>4	>8	>8	>4	>32	>4	>16	>8	>8	>64	>128	>8	>64
21-1160	Class B	-	-	-	>32	>16	>16	>2	≤1	>8	>8	>4	>32	>4	>16	>8	>8	32	>128	>8	>64
21-674	Class A	-	+	+	>32	>16	>16	>2	4	>8	>8	>4	32	>4	>16	8	>8	64	>128	>8	32
22-1133	Class A	-	-	+	>32	>16	>16	>2	≤1	>8	>8	>4	>32	>4	>16	>8	>8	32	>128	>8	>64
22-1134	Class B	+	-	-	>32	>16	>16	>2	≤1	>8	>8	>4	>32	>4	8	>8	>8	32	>128	>8	>64
22-1178	Class B	-	-	-	≤8	4	4	0.25	≤1	≤2	>8	≤1	16	>4	8	4	≤1	64	>128	≤2	8
22-1182	Class B	+	-	-	>32	>16	>16	>2	≤1	>8	>8	>4	>32	>4	2	>8	>8	32	>128	>8	>64
22-1189	Class A	-	-	+	>32	>16	>16	>2	≤1	>8	>8	>4	>32	>4	>16	>8	>8	32	>128	>8	>64
22-1202	CP	+	-	+	>32	>16	>16	>2	≤1	>8	>8	>4	>32	>4	>16	>8	>8	64	>128	>8	>64
22-1240	Class B	-	-	-	≤8	4	4	0.25	≤1	≤2	>8	≤1	16	>4	8	4	≤1	64	>128	≤2	8
22-1250	Class A	-	-	+	>32	>16	>16	>2	≤1	>8	>8	>4	>32	>4	>16	>8	>8	32	>128	>8	>64
22-1263	CP	-	+	-	>32	>16	>16	>2	≤1	>8	>8	>4	>32	>4	>16	>8	>8	64	>128	>8	16
22-1284	Class B	+	-	-	32	>16	>16	>2	≤1	4	>8	>4	16	>4	>16	>8	>8	>64	>128	>8	64
22-1285	Class D	-	-	-	>32	>16	>16	>2	≤1	>8	>8	>4	>32	>4	>16	>8	>8	>64	>128	>8	>64
22-1287	NR	+	-	-	≤8	8	16	1	≤1	≤2	>8	4	16	>4	>16	8	≤1	64	>128	4	16

22-1288	NR	-	-	-	8	16	8	1	≤1	4	8	4	16	>4	>16	8	2	>64	>128	4	16
22-1384	CP	-	-	+	>32	>16	>16	>2	≤1	>8	>8	>4	>32	>4	>16	>8	>8	>64	>128	>8	>64
22-1390	CP	-	+	-	>32	>16	>16	>2	≤1	>8	>8	>4	>32	>4	>16	>8	>8	>64	>128	>8	64
22-1395	Class A	-	-	+	>32	>16	>16	>2	≤1	>8	>8	>4	>32	>4	>16	>8	>8	32	>128	>8	>64
22-1397	CP	-	+	+	>32	>16	>16	>2	≤1	>8	>8	>4	>32	>4	>16	>8	>8	>64	>128	>8	16
22-1416	Class D	-	-	+	>32	>16	>16	>2	≤1	>8	>8	>4	>32	>4	≤1	>8	>8	32	>128	>8	>64
22-1420	Class A	-	-	+	>32	>16	>16	>2	≤1	>8	>8	>4	>32	>4	>16	>8	>8	32	>128	>8	>64
22-1428	CP	-	-	+	>32	>16	>16	>2	≤1	8	>8	>4	>32	>4	>16	>8	>8	>64	>128	>8	>64
22-1432	NR	-	-	+	8	8	4	1	≤1	4	>8	4	16	>4	16	4	≤1	64	>128	≤2	16
22-1442	Class D	+	-	-	≤8	2	4	1	2	>8	>8	2	>32	2	4	2	≤1	32	>128	≤2	≤4
22-1452	Class B	+	-	-	>32	>16	>16	>2	≤1	>8	>8	>4	>32	>4	8	>8	>8	64	>128	>8	64
22-1487	Class B	+	-	-	32	>16	>16	>2	≤1	4	>8	>4	>32	>4	>16	>8	>8	≤16	>128	>8	64
22-1491	NR	-	-	-	>32	>16	>16	>2	≤1	>8	>8	>4	16	>4	≤1	8	>8	32	>128	>8	64
22-1516	Class A	-	-	+	>32	4	8	0.5	≤1	4	>8	≤1	>32	>4	≤1	4	8	≤16	>128	≤2	32
22-1519	CP	-	+	-	>32	>16	>16	>2	≤1	>8	>8	>4	>32	>4	16	>8	>8	>64	>128	>8	32
22-1551	Class D	-	-	-	16	4	8	≤0.12	≤1	8	>8	≤1	16	>4	8	4	≤1	64	>128	≤2	8
22-1553	NR	-	-	-	32	4	8	≤0.12	≤1	4	>8	≤1	16	>4	8	4	≤1	64	>128	≤2	8
22-1555	Class D	-	-	-	>32	>16	>16	>2	≤1	>8	>8	>4	16	>4	16	8	>8	32	>128	>8	64
22-1558	Class A	-	-	+	>32	>16	>16	>2	≤1	>8	>8	>4	16	>4	16	8	>8	32	>128	>8	64
22-1573	Class B	+	-	-	>32	>16	>16	>2	≤1	>8	>8	>4	>32	>4	8	>8	>8	32	>128	>8	64
22-1574	Class D	-	-	-	32	4	4	≤0.12	≤1	4	>8	≤1	16	>4	8	2	≤1	64	>128	≤2	8
22-1579	Class D	-	-	-	32	4	4	≤0.12	≤1	8	>8	≤1	16	>4	8	2	≤1	64	>128	≤2	8
22-1580	Class D	-	-	-	32	4	4	≤0.12	≤1	8	>8	≤1	16	>4	8	2	≤1	32	>128	≤2	8
22-1590	Class D	-	-	-	16	4	4	≤0.12	≤1	8	>8	≤1	16	>4	8	2	≤1	64	>128	≤2	8
22-1593	NR	-	-	-	32	4	4	≤0.12	≤1	8	>8	≤1	16	>4	8	4	≤1	64	>128	≤2	8
22-1597	Class A	-	-	-	≤8	16	16	1	≤1	≤2	>8	4	32	>4	>16	8	≤1	>64	>128	4	32
22-1599	Class D	-	-	-	16	4	8	≤0.12	≤1	4	>8	≤1	16	>4	8	2	≤1	32	>128	≤2	8
22-1601	NR	-	-	-	32	4	4	≤0.12	≤1	8	>8	≤1	16	>4	8	2	≤1	64	>128	≤2	8
22-1609	Class D	-	-	-	32	4	4	≤0.12	≤1	4	>8	≤1	16	>4	8	2	≤1	64	>128	≤2	8
22-1614	Class B	+	-	-	>32	>16	>16	>2	≤1	>8	>8	>4	>32	>4	8	>8	>8	32	>128	>8	64
22-1618	NR	-	-	-	32	4	8	≤0.12	≤1	4	>8	≤1	16	>4	8	2	≤1	32	>128	≤2	8
22-1667	Class D	-	-	-	>32	4	16	0.25	≤1	8	>8	≤1	>32	>4	8	4	8	≤16	>128	≤2	32
22-1669	NR	-	-	-	32	4	4	>2	≤1	4	>8	>4	>32	>4	>16	2	>8	>64	>128	>8	8
22-1672	NR	-	-	-	≤8	4	4	0.25	≤1	≤2	>8	≤1	4	>4	8	2	≤1	≤16	>128	≤2	8
22-1673	NR	-	-	+	16	4	4	>2	≤1	4	>8	≤1	32	>4	8	4	≤1	64	>128	≤2	8
22-1675	NR	-	-	-	≤8	4	4	0.25	≤1	≤2	>8	≤1	8	>4	8	4	≤1	64	>128	≤2	8
22-1676	Class B	-	-	-	≤8	4	8	≤0.12	≤1	≤2	>8	≤1	8	>4	8	2	≤1	64	>128	≤2	32
22-1677	Class D	-	-	-	16	4	4	≤0.12	≤1	>8	>8	≤1	16	>4	8	2	≤1	64	>128	≤2	8
22-1678	NR	-	-	-	8	>16	>16	>2	≤1	4	8	>4	4	4	>16	>8	>8	>64	>128	>8	>64
22-1679	Class D	-	-	-	32	>16	>16	1	≤1	4	>8	2	>32	>4	16	2	≤1	32	>128	≤2	64
22-1680	Class B	-	+	-	>32	>16	>16	>2	≤1	>8	>8	>4	>32	>4	8	>8	>8	64	>128	>8	64
22-1681	Class B	+	-	-	>32	>16	>16	>2	≤1	>8	>8	>4	>32	>4	8	>8	>8	64	>128	>8	64
22-1682	Class B	+	-	-	16	>16	>16	0.25	≤1	4	>8	≤1	>32	>4	8	>8	>8	64	>128	≤2	>64
22-1683	CP	-	+	-	>32	>16	>16	>2	≤1	>8	>8	>4	>32	>4	>16	>8	>8	>64	>128	>8	32
22-1684	Class B	+	-	-	>32	>16	>16	>2	≤1	>8	>8	>4	>32	>4	8	>8	>8	64	>128	>8	64
22-1686	NR	-	-	+	>32	8	4	>2	≤1	4	>8	>4	>16	>4	16	4	≤1	>64	>128	>8	16
22-1687	NR	-	-	-	>32	>16	4	>2	≤1	4	>8	>4	>32	>4	>16	4	>8	>64	>128	>8	16
22-1689	NR	-	-	-	≤8	2	2	0.25	≤1	≤2	>8	≤1	8	>4	4	2	≤1	64	>128	≤2	≤4
22-1690	NR	-	-	-	≤8	2	2	0.25	≤1	≤2	>8	≤1	8	>4	8	2	≤1	>64	>128	≤2	≤4
22-1725	CP	-	+	-	>32	>16	>16	>2	≤1	>8	>8	>4	>32	>4	>16	>8	>8	>64	>128	>8	32
22-1756	CP	-	-	+	>32	>16	>16	>2	≤1	>8	>8	>4	>32	>4	>16	>8	>8	>64	>128	>8	>64
22-1758	NR	-	-	+	>32	>16	>16	>2	≤1	>8	>8	>4	>32	>4	>16	>8	>8	64	>128	>8	16
22-1759	NR	-	+	-	32	>16	>16	>2	≤1	>8	>8	>4	>32	>4	>16	>8	>8	64	>128	>8	32
22-1760	CP	-	-	+	≤8	>16	>16	>2	≤1	≤2	>8	>4	>32	>4	8	8	>8	64	>128	>8	64
22-1761	CP	-	+	-	16	>16	>16	>2	≤1	8	>8	>4	>32	>4	>16	>8	>8	>64	>128	>8	32
22-1762	NR	-	+	-	>32	>16	>16	>2	≤1	>8	>8	>4	>32	>4	>16	>8	>8	>64	>128	>8	16
22-1765	CP	-	-	+	>32	>16	>16	>2	≤1	>8	>8	>4	>32	>4	>16	>8	>8	>64	>128	>8	>64
22-1770	NR	+	-	-	32	16	>16	>2	≤1	4	>8	>4	32	>4	>16	>8	>8	≤16	>128	>8	64
22-1771	NR	-	-	-	≤8	2	>16	≤0.12	≤1	≤2	>8	≤1	>32	>4	8	2	≤1	>64	>128	≤2	≤4

AMK: amikacin, FEP: cefepime, CAZ: ceftazidime, CIP: ciprofloxacin, COL: colistin, GEN: gentamicin, IPM: imipenem, LVX: levofloxacin, MEM: meropenem, SXT: trimethoprim/sulfamethoxazole, ATM: aztreonam, CZA: ceftazidime-avibactam, CZT: ceftolozane/tazobactam, FOS: fosfomycin, NIT: nitrofurantoin, NOR: norfloxacin, TZP: piperacillin/tazobactam. NR: no recommendation, CP: carbapenemase producer. – negative, + positive
